# Effects of vitamin K administration on liver function, inflammation, and coagulation in chronic viral hepatitis: a retrospective study

**DOI:** 10.25122/jml-2026-0062

**Published:** 2026-04

**Authors:** Magdalena Lixandru, Ionela Maniu, Cosmin Ionuț Lixandru, Florin Grosu

**Affiliations:** 1 Faculty of Medicine, Lucian Blaga University, Sibiu, Romania; 2 Mathematics and Informatics Department, Faculty of Sciences, Research Center in Informatics and Information Technology, Lucian Blaga University, Sibiu, Romania; 3 Pediatric Clinical Hospital Sibiu, Sibiu, Romania

**Keywords:** vitamin K, chronic viral hepatitis, inflammation, coagulation, liver function, ALT, alanine aminotransferase, AST, aspartate aminotransferase, CRP, C-reactive protein, ESR, erythrocyte sedimentation rate, INR, international normalized ratio, PT, prothrombin time, aPTT, activated partial thromboplastin time, IQR, interquartile range, HBsAg, hepatitis B surface antigen, HCV, hepatitis C virus, HDV, hepatitis D virus, HEV, hepatitis E virus, HAV, hepatitis A virus, IL-6, interleukin-6, TNF-α, tumor necrosis factor alpha, NF-κB, nuclear factor kappa B

## Abstract

Vitamin K is essential for coagulation and has increasingly been recognized for its potential role in inflammatory processes. In patients with chronic viral hepatitis, alterations in coagulation parameters and persistent systemic inflammation are frequently observed. This study aimed to evaluate the association between vitamin K administration and changes in liver enzymes, inflammatory markers, and coagulation parameters in patients with chronic viral hepatitis. This retrospective observational study included 94 patients with chronic viral hepatitis hospitalized between January 2020 and December 2024. Laboratory parameters reflecting liver function, inflammatory status, and coagulation profile were assessed at admission and discharge following vitamin K administration. Changes in biological parameters were analyzed using non-parametric tests for paired data. Reductions in liver enzymes, inflammatory markers, and coagulation parameters were observed between admission and discharge. Aspartate aminotransferase and alanine aminotransferase levels decreased, accompanied by lower values of C-reactive protein and erythrocyte sedimentation rate. Coagulation parameters, including prothrombin time and international normalized ratio, also decreased. No significant differences were identified between patients with and without hepatitis B infection. Vitamin K administration in patients with chronic viral hepatitis was associated with changes in liver enzymes, inflammatory markers, and coagulation parameters. These findings suggest a potential role for vitamin K in the interplay between inflammation and hemostasis in chronic liver disease, while its clinical utility should be considered on an individual basis.

## Introduction

Vitamin K is an essential fat-soluble vitamin required as a cofactor for γ-glutamyl carboxylase, an enzyme responsible for the post-translational γ-carboxylation of vitamin-K-dependent proteins, including the hepatic coagulation factors II, VII, IX, and X, as well as proteins C and S [1]. This carboxylation step enables calcium binding and is indispensable for the biological activity of these proteins, making vitamin K fundamental for normal hemostasis [1,2]. In chronic liver disease, impaired bile secretion, reduced intestinal absorption of fat-soluble vitamins, and compromised hepatic metabolic function frequently contribute to decreased vitamin K availability, predisposing patients to functional vitamin K deficiency and coagulation abnormalities [3].

Chronic viral hepatitis, particularly hepatitis B and C, is characterized by persistent hepatic inflammation, progressive hepatocellular injury, and dysregulation of both synthetic and metabolic liver functions [4]. Ongoing necroinflammation alters hepatocyte protein synthesis and frequently manifests as abnormalities in coagulation parameters, including prolonged prothrombin time (PT)/international normalized ratio (INR), even in the absence of clinically relevant bleeding risk [3]. Beyond impairing hepatic synthetic capacity, chronic viral hepatitis is also associated with elevated systemic inflammatory markers, such as C-reactive protein (CRP) and erythrocyte sedimentation rate (ESR), which correlate with disease activity and fibrosis progression [5]. Emerging observational evidence suggests that vitamin K status may be inversely associated with systemic inflammation, with higher circulating phylloquinone levels being linked to lower concentrations of inflammatory biomarkers such as interleukin-6 (IL-6) and CRP [6]. These findings raise the possibility that vitamin K administration may exert beneficial effects not only on coagulation parameters but potentially also on inflammatory pathways in patients with chronic liver disease, including those with chronic viral hepatitis [6].

Despite its frequent use in clinical practice, the effectiveness of vitamin K administration in correcting coagulation abnormalities in chronic liver disease remains controversial, as several studies have shown minimal or no improvement in INR following supplementation, suggesting that prolonged INR in liver disease often reflects impaired synthesis of coagulation factors rather than true vitamin K deficiency [7]. Moreover, the concept of “rebalanced hemostasis” in chronic liver disease indicates that routine coagulation tests poorly reflect actual bleeding risk, further questioning the value of empiric vitamin K administration in this setting [8]. Nonetheless, functional vitamin K deficiency can occur in subgroups of patients with chronic liver disease—particularly those with cholestasis, malnutrition, or significant inflammatory burden—which raises the possibility that targeted vitamin K administration may offer benefits beyond coagulation correction, including modulation of inflammatory activity [9]. However, data specifically assessing the effects of vitamin K on liver function parameters and inflammatory biomarkers in patients with chronic viral hepatitis are extremely limited, thereby representing a significant gap in the literature that warrants further investigation.

Beyond its established role in coagulation, vitamin K has increasingly been recognized for its potential anti-inflammatory effects. Observational studies in community-based populations have demonstrated inverse associations between dietary vitamin K intake and circulating levels of inflammatory biomarkers, including CRP and IL-6, suggesting that higher vitamin K status may be linked to a more favorable systemic inflammatory profile [10]. Experimental data further support these findings: menaquinone-4 (vitamin K2) has been shown to suppress lipopolysaccharide-induced production of pro-inflammatory cytokines such as tumor necrosis factor alpha (TNF-α) and IL-6 through inhibition of the nuclear factor kappa B (NF-κB) pathway in human macrophage models [11]. Given that chronic viral hepatitis is characterized by persistent hepatic and systemic inflammation, these mechanistic and epidemiologic observations raise the possibility that vitamin K may have clinically relevant immunomodulatory effects in patients with chronic liver disease, although this hypothesis has not been adequately examined in clinical settings.

Despite mechanistic and observational evidence suggesting that vitamin K status may influence both coagulation and inflammatory processes, clinical studies evaluating the effects of vitamin K administration in chronic liver disease have produced inconsistent results, with most data derived from heterogeneous populations predominantly consisting of patients with advanced cirrhosis rather than those with chronic viral hepatitis [7,9]. Moreover, most available studies have assessed only coagulation parameters—typically INR—without examining broader markers of liver function or systemic inflammation, thereby overlooking potentially relevant biochemical responses to vitamin K supplementation [12]. Considering that chronic viral hepatitis represents a distinct pathophysiological entity characterized by ongoing hepatocellular inflammation, fluctuating liver function, and variable synthetic capacity, it remains unclear whether vitamin K administration may differentially affect hepatic biochemical profiles or inflammatory markers in this subgroup of patients [4]. This evidence gap highlights the need for targeted research evaluating the biochemical effects of vitamin K administration in individuals with chronic viral hepatitis.

The primary objective of this study was to assess the biochemical and clinical effects of vitamin K administration in patients with chronic viral hepatitis. Using a retrospective evaluation of laboratory data obtained before and after supplementation, the study aims to determine whether vitamin K contributes to measurable improvements in hepatic function and inflammatory status in this population. By clarifying the potential therapeutic role of vitamin K in chronic viral hepatitis, the findings may help inform and guide more evidence-based clinical decision-making regarding its use in routine practice.

## Material and Methods

This retrospective observational study was conducted in the Internal Medicine Department of Valcea County Emergency Hospital over 5 years, from January 2020 to December 2024. Patients were eligible for inclusion if they met the following criteria: a confirmed diagnosis of chronic viral hepatitis established by positive serological testing; age between 18 and 65 years; and a clinically stable condition at the time of hospitalization, without evidence of recent acute complications. All included patients provided written informed consent for the use of their medical data for research purposes. In addition, only patients with available and measurable laboratory data reflecting hepatic function and inflammatory status were considered eligible for analysis. To ensure reliable assessment of treatment response and disease progression, only patients with sufficient clinical and laboratory data recorded during hospitalization were included in the final analysis.

Patients were excluded if they had other significant liver diseases, including advanced liver cirrhosis, or major comorbid conditions likely to interfere with study outcomes, such as severe renal impairment, hepatocellular carcinoma, or active systemic infections or septic states. Patients receiving oral anticoagulant therapy or medications known to interfere with vitamin K metabolism were also excluded. In addition, patients undergoing immunosuppressive treatment that could significantly influence immune response and inflammatory markers were excluded, as such therapies might confound the interpretation of study results. Pregnant or breastfeeding women were excluded due to potential risks to the fetus or infant and the possible influence of hormonal changes on study outcomes. Finally, patients enrolled in other clinical studies involving pharmacological interventions that could interfere with the objectives of the present study were excluded to minimize confounding. Among the 94 patients included in the study, 72 received vitamin K intravenously, while the remaining 22 received it orally, with a dose of 10 mg in both routes. The choice of administration route was based on clinical judgment and routine hospital practice, with intravenous administration generally preferred in patients with more severe coagulation abnormalities or impaired oral intake. In contrast, oral administration was used in clinically stable patients. The median hospitalization duration was 10 days (IQR 7–14 days).

Data were presented using median values and interquartile ranges (IQR: (25^th^ and 75^th^ percentiles) or mean and standard deviation in case of continuous variables, and as frequencies and percentages in case of categorical variables. To compare different biological parameters before and after vitamin K administration, nonparametric tests for paired data (Friedman or Wilcoxon) were used. A *P* value < 0.05 was considered statistically significant.

## Results

In the final analysis, this study included 94 patients with chronic viral hepatitis. Baseline demographic, clinical, and virological characteristics of the study population are presented in Table 1. The study population was predominantly male (61%), with a mean age of 62.00 years. Patients were equally distributed between urban and rural areas. Regarding viral etiology, 43% of patients were positive for hepatitis B and 38% for hepatitis C, while hepatitis D co-infection was identified in 18% of cases. A smaller proportion of patients presented markers of hepatitis E (7.4%) and acute hepatitis A (26%).

**Table 1:** Baseline characteristics of the study population

Characteristic	*n* = 94
Patient sex	
Male	57 (61%)
Female	37 (39%)
Patient age	61.31 (±15.02)
Area of residence	
Urban	47 (50%)
Rural	47 (50%)
Ag Hbs (Hepatitis B)	40 (43%)
Ac HCV (Hepatitis C)	36 (38%)
Ag HVD (Hepatitis D)	17 (18%)
Ac HEV (Hepatitis E Western blot)	7 (7.4%)
Anti-HAV-IgM (Hepatitis A)	24 (26%)
Smoker	40 (43%)
Alcohol consumption	45 (48%)
Death	31 (33%)
Hospitalization	10.00 (7.00, 14.00)

Lifestyle-related factors showed that 43% of patients were smokers and 48% reported alcohol consumption. The in-hospital mortality rate was 33.0%, and the median hospitalization duration was 10.00 days.

Laboratory parameters reflecting liver function, inflammatory status, and coagulation profile were assessed at admission and discharge to evaluate changes associated with vitamin K administration. Subsequent analyses compared baseline and post-treatment values and examined subgroup differences by viral infection status.

Changes in liver transaminase levels before and after vitamin K administration are presented in Table 2. A statistically significant decrease in both transaminase levels was observed. Median aspartate aminotransferase (AST) values decreased from 43.00 (27.00–76.00) at admission to 36.50 (21.00–68.00) at discharge (*P* < 0.001). Similarly, alanine aminotransferase (ALT) values decreased from 29.00 (19.00–55.00) to 27.00 (12.00–50.00) (*P* < 0.001).

**Table 2:** Changes in liver transaminases before and after vitamin K administration

Characteristic	Admission	Discharge	*P* value
AST	43.00 (27.00, 76.00)	36.50 (21.00, 68.00)	0.000
ALT	29.00 (19.00, 55.00)	27.00 (12.00, 50.00)	0.000

AST, aspartate aminotransferase; ALT, alanine aminotransferase

Changes in inflammatory markers following vitamin K administration are presented in Table 3. A statistically significant decrease in both inflammatory markers was observed during hospitalization. Median CRP levels decreased from 14.40 (8.60–24.00) at admission to 8.00 (5.10–12.60) at discharge (*P* < 0.001). Likewise, ESR values decreased from 40.00 (30.00–50.00) to 18.00 (12.00–34.00) (*P* < 0.001).

**Table 3:** Changes in inflammatory markers before and after vitamin K administration

Characteristic	Admission	Discharge	*P* value
CRP	14.40 (8.60, 24.00)	8.00 (5.10, 12.60)	0.000
ESR	40.00 (30.00, 50.00)	18.00 (12.00, 34.00)	0.000

Changes in coagulation parameters following vitamin K administration are presented in Table 4. A statistically significant reduction in coagulation parameters was observed, and median prothrombin time (PT) decreased from 13.00 (11.00–14.00) at admission to 11.35 (11.00–13.00) at discharge (*P* < 0.001). In addition, INR values decreased from 1.95 (1.50–2.70) to 1.80 (1.30–2.20) (*P* < 0.001).

**Table 4:** Changes in coagulation parameters before and after vitamin K administration

Characteristic	Admission	Discharge	*P* value
PT	13.00 (11.00, 14.00)	11.35 (11.00, 13.00)	0.000
INR	1.95 (1.50, 2.70)	1.80 (1.30, 2.20)	0.000
APTT	33.25 (31.00, 37.00)	32.00 (29.00, 34.00)	0.000
Fibrinogen	329.00 (265.00, 450.00)	314.00 (245.00, 400.00)	0.000

PT, Median prothrombin time; INR, International normalized ratio; APTT, Activated partial thromboplastin time

Activated partial thromboplastin time (aPTT) showed a decrease from 33.25 (31.00–37.00) to 32.00 (29.00–34.00) (*P* < 0.001), while fibrinogen levels decreased from 329.00 (265.00–450.00) to 314.00 (245.00–400.00) (*P* < 0.001).

Comparison of laboratory parameters according to hepatitis B (HBsAg) status is presented in Table 5. No statistically significant differences were observed between patients with and without hepatitis B infection in liver function tests, inflammatory markers, or coagulation parameters, either at admission or at discharge (all *P* > 0.05).

**Table 5:** Comparison of laboratory parameters according to hepatitis B (HBsAg) status

Characteristic	WithoutHBsAg (Hepatitis B)*n* = 54		WithHBsAg (Hepatitis B)*n* = 40		*P* value	
	Admission	Discharge	Admission	Discharge	*	**
AST	48.50 (26.00, 78.00)	40.00 (21.00, 69.00)	37.50 (28.00, 61.50)	33.00 (21.50, 60.00)	0.4	0.4
ALT	37.00 (22.00, 61.00)	33.00 (18.00, 54.00)	26.00 (16.50, 46.00)	21.50 (11.00, 42.00)	0.093	0.090
CRP	13.45 (9.00, 23.70)	7.95 (5.50, 11.00)	15.85 (7.30, 27.00)	8.95 (4.00, 14.00)	>0.9	0.5
ESR	40.00 (29.00, 50.00)	18.50 (13.00, 34.00)	40.00 (34.00, 49.00)	18.00 (12.00, 33.50)	>0.9	0.7
INR	2.20 (1.70, 2.70)	1.85 (1.40, 2.40)	1.90 (1.40, 2.70)	1.70 (1.22, 2.20)	0.3	0.2

* *P* values for comparisons between patients without HBsAg (hepatitis B) at admission vs. patients with HBsAg (hepatitis B) at admission; **P values for comparisons between patients without HBsAg (Hepatitis B) at discharge vs. patients with HBsAg (Hepatitis B) at discharge AST, aspartate aminotransferase; ALT, alanine aminotransferase; CRP, Median C-reactive protein; ESR, Erythrocyte sedimentation rate; INR, International normalized ratio

## Discussion

This study evaluated the biochemical effects of vitamin K administration in patients with chronic viral hepatitis, focusing on liver function tests, inflammatory markers, and coagulation parameters. The main findings include reductions in transaminases, inflammatory markers, and coagulation parameters observed between admission and discharge.

AST and ALT levels decreased between admission and discharge. Although vitamin K is not typically considered to have a direct hepatoprotective effect, this finding may reflect reduced hepatic inflammatory activity. Chronic viral hepatitis is characterized by persistent immune-mediated hepatocellular injury, which is commonly associated with elevated transaminase levels [4]. Therefore, the observed reduction in liver enzymes may reflect a decrease in inflammatory activity during hospitalization rather than a direct pharmacological effect of vitamin K. In addition, supportive care and the natural course of the disease may have contributed to these changes, and the results should be interpreted with caution. Because this was a retrospective observational study conducted during hospitalization, the observed biochemical improvements cannot be attributed exclusively to vitamin K administration. Supportive measures routinely provided during hospitalization, including intravenous fluids, nutritional support, treatment of concomitant conditions, monitoring, and general medical care, may also have contributed to the improvement in liver function tests, inflammatory markers, and coagulation parameters. In addition, spontaneous fluctuations in disease activity and the natural course of chronic viral hepatitis may have influenced the observed changes. Therefore, the results should be interpreted with caution, and a direct causal relationship between vitamin K administration and biochemical improvement cannot be definitively established. Information regarding antiviral or other concomitant therapies administered during hospitalization was not systematically evaluated in the present study. Therefore, their potential influence on liver function tests, inflammatory markers, and coagulation parameters cannot be excluded.

In addition to liver enzymes, a reduction in inflammatory markers, including CRP and ESR, was observed. These findings are consistent with previous observational and experimental studies suggesting a potential anti-inflammatory role of vitamin K. Higher vitamin K intake has been associated with lower circulating levels of inflammatory biomarkers, such as CRP and interleukin-6 [11]. Furthermore, experimental studies have demonstrated that vitamin K can suppress the production of pro-inflammatory cytokines by inhibiting the NF-κB signaling pathway [12]. Although the present study did not directly assess cytokine levels, the observed decreases in CRP and ESR support the hypothesis that vitamin K may modulate systemic inflammation in patients with chronic liver disease.

One of the most relevant findings of this study was the reduction in INR and prothrombin time observed between admission and discharge. This finding appears to contrast with several previous studies reporting minimal or no improvement in coagulation parameters following vitamin K administration in patients with chronic liver disease [8]. In these studies, prolonged INR has been interpreted primarily as a consequence of impaired hepatic synthesis of clotting factors rather than true vitamin K deficiency, in the context of the so-called “rebalanced hemostasis” [9]. However, the improvement observed in the present study may suggest a functional vitamin K deficiency in a subset of patients, potentially related to impaired absorption, nutritional status, or an increased inflammatory burden [10].

Another possible explanation for the observed improvement in coagulation parameters is that vitamin K supplementation may be more effective in patients with partially preserved hepatic synthetic function. Previous studies have suggested that the response to vitamin K in liver disease is heterogeneous and may depend on the underlying severity of hepatic dysfunction and the presence of true or functional vitamin K deficiency. For instance, Rivosecchi *et al*. reported that vitamin K administration in critically ill patients with liver-related coagulopathy was associated with modest improvements in INR in selected cases, highlighting the variability of response [13]. Similarly, Saja *et al*. emphasized that vitamin K may be beneficial in patients with reversible causes of coagulopathy, while having a limited impact in advanced liver failure [14]. In this context, the changes observed in our study may reflect a reversible component of coagulation impairment rather than irreversible hepatic dysfunction.

Our findings should also be interpreted in the context of previous clinical studies evaluating the effect of vitamin K on coagulation abnormalities in liver disease. In contrast to our results, several studies have reported minimal or no improvement in INR following vitamin K administration. For example, Meyer *et al*. found that vitamin K supplementation did not significantly reduce INR values or bleeding risk in patients with cirrhosis [15]. Similarly, Al Sulaiman *et al*. reported that although INR decreased after vitamin K administration in critically ill patients, this effect was not associated with improved clinical outcomes [16]. These findings suggest that changes in standard coagulation parameters may not necessarily reflect clinically meaningful improvements in hemostasis. In this context, the improvement observed in our study may be due to a reversible component of coagulopathy or to differences in patient characteristics and disease severity.

It is important to note that standard coagulation parameters, particularly INR, may not accurately reflect bleeding risk in patients with chronic liver disease. The concept of “rebalanced hemostasis” suggests that although both procoagulant and anticoagulant factors are reduced, a new equilibrium is established, making conventional coagulation tests insufficient for predicting clinical bleeding [9]. Previous studies have shown a poor correlation between INR values and actual bleeding risk in patients with liver disease, further questioning the clinical significance of isolated INR changes [17]. Therefore, although a statistically significant reduction in INR was observed in our study, its direct clinical relevance should be interpreted with caution.

In the present study, subgroup analysis according to hepatitis B status did not reveal significant differences in liver function tests, inflammatory markers, or coagulation parameters between patients with and without HBV infection. This finding suggests that the observed biochemical changes were not dependent on viral etiology and may reflect common pathophysiological mechanisms shared across different forms of chronic viral hepatitis. Chronic hepatitis B and C infections are both characterized by persistent immune-mediated inflammation and hepatocellular injury, which may lead to similar alterations in liver function and systemic inflammatory profiles [4]. Therefore, the lack of significant differences between subgroups supports the generalizability of the observed effects across patients with chronic viral hepatitis.

Several limitations of this study should be acknowledged. First, the retrospective design limits the ability to establish a causal relationship between vitamin K administration and the observed biochemical changes. Second, the relatively small sample size may reduce the statistical power, particularly in subgroup analyses. Third, baseline vitamin K levels were not measured, making it difficult to distinguish between true vitamin K deficiency and functional impairment. In addition, the absence of a control group limits the ability to differentiate the effects of vitamin K administration from the natural course of the disease or other supportive treatments. Furthermore, the short-term evaluation limited to the hospitalization period does not allow assessment of long-term outcomes. Finally, the lack of clinical endpoints, such as bleeding events, limits the interpretation of the clinical relevance of the observed biochemical changes.

Taken together, the findings of the present study indicate that vitamin K administration in patients with chronic viral hepatitis is accompanied by measurable changes in liver enzymes, inflammatory markers, and coagulation parameters. While these observations may reflect a potential role of vitamin K in modulating biochemical pathways related to inflammation and hemostasis, they should be interpreted in the context of the study design and the presence of potential confounding factors.

## Conclusion

Vitamin K administration in patients with chronic viral hepatitis was associated with measurable changes in liver enzymes, inflammatory markers, and coagulation parameters during hospitalization. These findings suggest a potential role of vitamin K in the interplay between inflammation and hemostasis in chronic liver disease, beyond its traditional function in coagulation.

From a clinical perspective, vitamin K may be useful in selected patients, particularly when a reversible component of coagulopathy is present, while its routine use should be carefully considered.

## Data Availability

Further data is available from the corresponding author upon reasonable request.
